# Advancements in Diagnosis and Management of Distal Radioulnar Joint Instability: A Comprehensive Review Including a New Classification for DRUJ Injuries

**DOI:** 10.3390/jpm14090943

**Published:** 2024-09-05

**Authors:** Awad Dmour, Stefan-Dragos Tirnovanu, Dragos-Cristian Popescu, Norin Forna, Tudor Pinteala, Bianca-Ana Dmour, Liliana Savin, Bogdan Veliceasa, Alexandru Filip, Adrian Claudiu Carp, Paul Dan Sirbu, Ovidiu Alexa

**Affiliations:** 1 Department of Orthopedics and Traumatology, Faculty of Medicine, “Grigore T. Popa” University of Medicine and Pharmacy, 700115 Iasi, Romania; awad.dmour@d.umfiasi.ro (A.D.); dragos.popescu@umfiasi.ro (D.-C.P.); norin.forna@umfiasi.ro (N.F.); tudor_pinteala@umfiasi.ro (T.P.); liliana.savin@umfiasi.ro (L.S.); alexandru-filip@umfiasi.ro (A.F.); adrian-claudiu.carp@umfiasi.ro (A.C.C.); paul.sirbu@umfiasi.ro (P.D.S.); ovdiu.alexa@umfiasi.ro (O.A.); 2 Department of Orthopaedics and Traumatology, “Sf. Spiridon” Emergency Universitary Hospital, 700115 Iasi, Romania; 3 Department Orthopedics and Traumatology, Clinical Rehabilitation Hospital, 700661 Iasi, Romania; 4 Department of Internal Medicine, “Grigore T. Popa” University of Medicine and Pharmacy, 700115 Iasi, Romania; bianca-ana.gherasim-dmour@d.umfiasi.ro; 5 Department of III Internal Medicine Clinic, “St. Spiridon” County Clinical Emergency Hospital, 700111 Iasi, Romania

**Keywords:** distal radioulnar joint (DRUJ) instability, distal radioulnar joint (DRUJ) classification, triangular fibrocartilage complex (TFCC) injuries, imaging modalities, DRUJ arthroscopy

## Abstract

Distal radioulnar joint (DRUJ) instability is a complex condition that can severely affect forearm function, causing pain, limited range of motion, and reduced strength. This review aims to consolidate current knowledge on the diagnosis and management of DRUJ instability, emphasizing a new classification system that we propose. The review synthesizes anatomical and biomechanical factors essential for DRUJ stability, focusing on the interrelationship between the bones and surrounding soft tissues. Our methodology involved a thorough examination of recent studies, incorporating clinical assessments and advanced imaging techniques such as MRI, ultrasound, and dynamic CT. This approach allowed us to develop a classification system that categorizes DRUJ injuries into three distinct grades. This system is intended to be practical for both clinical and radiological evaluations, offering clear guidance for treatment based on injury severity. The review discusses a range of treatment options, from conservative measures like splinting and physiotherapy to surgical procedures, including arthroscopy and DRUJ arthroplasty. The proposed classification system enhances the accuracy of diagnosis and supports more effective decision making in clinical practice. In summary, our findings suggest that the integration of advanced imaging techniques with minimally invasive surgical interventions can lead to better outcomes for patients. This review serves as a valuable resource for clinicians, providing a structured approach to managing DRUJ instability and improving patient care through the implementation of our new classification system.

## 1. Introduction

The distal radioulnar joint (DRUJ) is a critical component of the forearm, enabling pronation and supination actions necessary for functional movements. It consists of the radius, which serves as the mobile portion of the distal part of the forearm, rotating around the ulna. The DRUJ, in conjunction with the proximal radioulnar joint, acts as a unified mechanism responsible for forearm rotation [1].

Ensuring the stability of the DRUJ is essential for maintaining optimal forearm function. This stability is maintained by the integrity of both the bones and the surrounding soft tissues, including muscles and ligaments that attach to the joint [2]. Disruptions in either the bony structures or the soft tissues can lead to joint instability, resulting in pain, restricted range of motion, and functional impairments.

Joint instability and arthritis are common pathologies that can affect the DRUJ. These conditions may occur independently or in combination, significantly impacting patient well-being [3]. Arthritis involves inflammation and degeneration of the joint, leading to pain, stiffness, and reduced joint function. Joint instability, on the other hand, refers to excessive joint movement or laxity, causing instability and functional limitations.

In this review article, we aim to provide a comprehensive analysis of the DRUJ, encompassing its anatomy, function, and various pathologies. Drawing from the latest research and clinical insights, we explore the mechanisms of DRUJ stability, highlighting the intricate interplay between bones and soft tissues. Furthermore, we delve into the etiology, clinical presentation, diagnostic approaches, and treatment modalities for DRUJ pathology.

By enhancing our understanding of the DRUJ and its associated pathologies, we can improve diagnosis, treatment, and patient outcomes. This review article aims to serve as a valuable resource for healthcare professionals, researchers, and individuals interested in the field of upper-limb orthopedics.

## 2. Anatomy of the Distal Radioulnar Joint

Understanding the anatomy of the DRUJ is vital for evaluating and diagnosing conditions like DRUJ instability, triangular fibrocartilage complex (TFCC) tears, and fractures. The DRUJ consists of the distal radius (specifically, the ulnar notch) and the ulnar head, the radius of curvature of which is smaller by approximately 4–7 mm [3,4]. Achieving stability in this joint requires a delicate balance between the bones, contributing to around 20% of stability, and the soft tissues, which provide passive and active stability [4,5].

The ulnar notch covers 130°, and the ulnar head covers 200° of joint surfaces with cartilage, allowing for necessary translational and rotational movements for pronation and supination [6]. The fovea is a small depression on the ulnar head where the foveal ligament attaches; it is an important stabilizer of the DRUJ and is often injured in TFCC tears [7]. The ulnar styloid, a bony projection continuing distally from the dome of the ulna, features a shallow concavity known as the fovea, which serves as a critical area for soft tissue attachment [8].

The vascular supply to the TFC (triangular fibrocartilage) is primarily derived from the ulnar artery through its palmar and dorsal radiocarpal branches, as well as the dorsal branch of the anterior interosseous artery and the palmar branch of the anterior interosseous artery. These vessels are arranged radially in relation to the fibrocartilage. Histological examinations of the TFC demonstrate vascularity in the outer 15% to 20% of the disc, while the rest of the structure is avascular. The branches originating from the ulnar artery play a significant role in supplying blood to the TFC region of the complex [9].

The interosseous membrane (IOM) serves as a delicate, fibrous structure that acts as a barrier between the radius and ulna bones in the forearm. Injuries to the IOM can arise from various factors, such as trauma, infections, or inflammatory conditions. These lesions can give rise to symptoms such as discomfort, diminished strength, and instability within the forearm [10].

**Figure 1:**
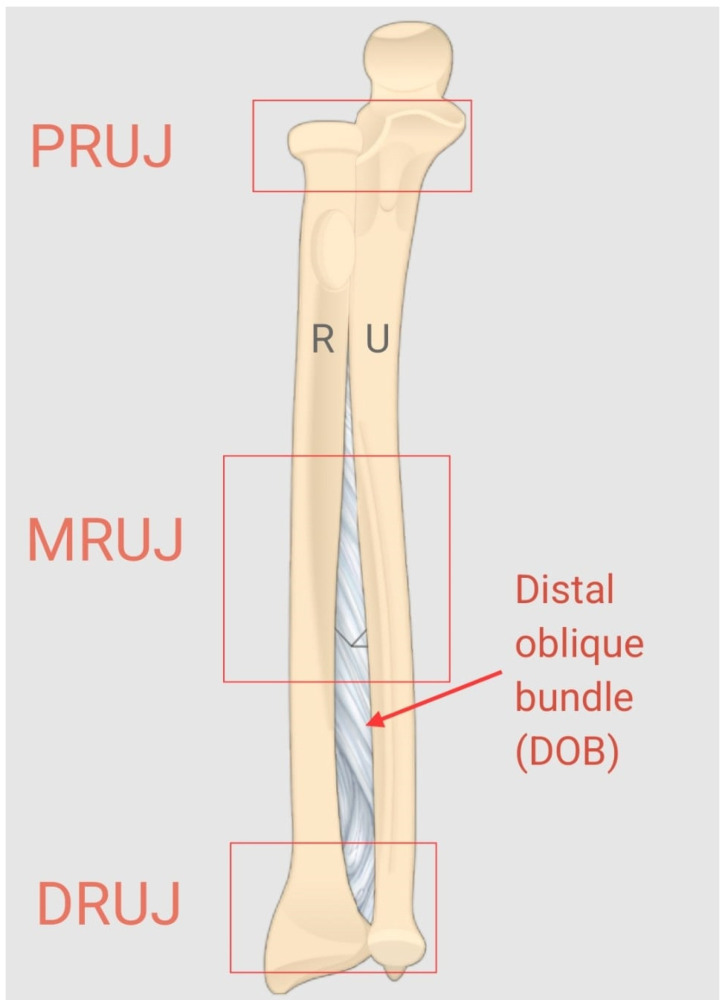
Anatomy of the radius (R) and ulna (U) showing the DOB and illustrating of the three-locker system concept.

The three-locker system classifies forearm joint injuries by focusing on the proximal radioulnar joint (PRUJ), mid-radioulnar joint (MRUJ), and distal radioulnar joint (DRUJ) (Figure 1). It categorizes injuries into three types based on the extent of damage to key structures like the interosseous membrane, the TFCC, and the forearm bones. This system helps in understanding the anatomical impact across the entire radioulnar joint complex and guides appropriate treatment strategies [11].

In conclusion, a thorough understanding of DRUJ anatomy is fundamental for clinicians managing wrist and forearm conditions, enabling accurate diagnoses and effective treatment plans tailored to each patient’s requirements.

## 3. Biomechanics of the Distal Radioulnar Joint (DRUJ)

Motion at the DRUJ is not purely axial but combines anterior-to-posterior translation with proximal-to-distal translation [12]. The joint allows pronation–supination movements of approximately 150° and ensures stability during joint translation [4,5]. Axial loading with wrist hyperextension and forearm pronation can cause separation between the ulna and radius at the DRUJ, leading to changes in ulnar translation and rotation [13]

Positive ulnar variance, indicating a longer distal ulna, can result in increased load transmission at the ulnocarpal joint and increase the likelihood of ulnar impaction or ulnocarpal abutment syndrome [14]. The relative length index between the distal ulna and the distal radius, known as ulnar variance or Hulten’s variance, determines whether the ulna is distally longer (positive ulnar variance, when the level of the ulna is >2.5 mm beyond the radius margin at the distal radioulnar joint) or shorter (negative ulnar variance, when the ulna is ≤2.5 mm than the radius at the DRUJ) [4,9]. This variance in ulnar length relative to the radius can have implications for the stability and biomechanics of the wrist joint.

Soft tissues (both static and dynamic) contribute to DRUJ stability. Static structures such as the TFCC, the dorsal and volar radio ulnar ligaments, the collateral ulnar ligament, and the joint capsule provide stability. Dynamic muscle stabilizers include the extensor carpi ulnaris tendon and the pronator quadratus muscle [4]. The TFCC and IOM collectively provide 70% to 80% of the total stability during forearm rotation [3].

The distal oblique bundle (DOB), part of the distal portion of the IOM, plays a crucial role in providing stability to the entire forearm [15]. Additionally, the distal IOM serves as an important secondary stabilizer of the DRUJ [16]. In the context of a distal radius fracture, where the TFCC is frequently injured, the usually intact DIOM plays a central role in maintaining DRUJ stability [16].

The three-locker system also helps in assessing the biomechanical effects of DRUJ injuries by linking specific anatomical disruptions to changes in joint stability and function. By categorizing injuries, it offers a clearer understanding of their biomechanical impact, assisting in both surgical and non-surgical treatment decisions [11].

Understanding the intricate anatomy and biomechanics of the DRUJ is crucial for diagnosing and managing various pathologies affecting this joint. It helps in selecting the best therapeutic options for forearm injuries that often involve bone fractures with joint instability [17].

## 4. Clinical Evaluation

The diagnosis of DRUJ instability requires a thorough clinical examination and specific tests. Wrist examination is crucial for assessing DRUJ instability, particularly in patients with ligament laxity [1,3]. Careful inspection of both fists can reveal signs such as protrusion of the ulnar head, indicating dorsal displacement relative to the radius, or a dimple instead of the ulnar head, suggesting volar displacement [1,18].

In the acute setting, assessing the stability of the DRUJ involves various maneuvers. With the patient’s forearm in a neutral position and the elbow stabilized, the amount of volar and dorsal translation of the distal ulna is evaluated while stabilizing the radius. Pronation and supination are also tested, with the ulna pushed toward the patient to assess the firmness of the end point and elicit any pain. Comparing these findings to those on the contralateral side is important, and audible or palpable clinking may occur during active and passive pronation and supination. Obvious subluxation of the ulnar head indicates DRUJ instability [18,19].

The DRUJ ballottement test is essential in clinical practice for evaluating the stability of the joint. It involves applying pressure to the ulnar head while stabilizing the radius to assess the mobility of the ulnar head relative to the radius. Typically performed with the forearm in a neutral position, this maneuver evaluates both active and passive pronation and supination to further gauge DRUJ stability. Recent studies utilizing advanced techniques like three-dimensional electromagnetic tracking devices have underscored the test’s reliability and validity by demonstrating a significant correlation between measured DRUJ movement and clinical instability [20]. Despite variations in predictive values and sensitivity noted in studies involving specialist hand surgeons [21], the DRUJ ballottement test remains indispensable for the diagnosis of DRUJ instability and in guiding treatment decisions in clinical settings. Ongoing research and standardization efforts aim to improve the test’s reliability and validity, potentially leading to more accurate diagnoses and better patient outcomes [20,21,22].

Chronic cases benefit from specific diagnostic tests, such as the ulnar fovea sign, which elicits pain between the ulnar styloid and the flexor tendon of the carpi ulnaris, indicative of ligament involvement [3,19]. The dorso-palmar stress test assesses instability based on the degree of ulnar head translation during firm radius stabilization [23]. Additionally, the press test, where ulno-carpal pain upon rising from an armchair suggests possible TFCC lesions, is also valuable [23,24].

The Beighton score serves as a screening tool for joint hypermobility, evaluating various joints to assess hypermobility-related issues alongside DRUJ-specific tests [13]. Integration of these tests provides a thorough evaluation of DRUJ function, facilitating accurate diagnosis and guiding appropriate treatment decisions.

## 5. Imaging of the DRUJ

Assessing DRUJ pathology and determining suitable treatment options may require a multimodal imaging approach due to the complex anatomy and small structures involved. Obtaining clear images of the DRUJ can be challenging due to its intricate nature [25]. A comprehensive evaluation should encompass static, dynamic, and functional relationships, as well as abnormalities affecting the bones, ligaments, and muscles within the joint [26]. The integration of automated methods addresses the limitations of manual two-dimensional measurements, leading to reduced inter-observer variability and improved accuracy in diagnosing and planning treatment for patients with DRUJ pathologies [27].

### 5.1. Conventional Radiography

**Figure 2:**
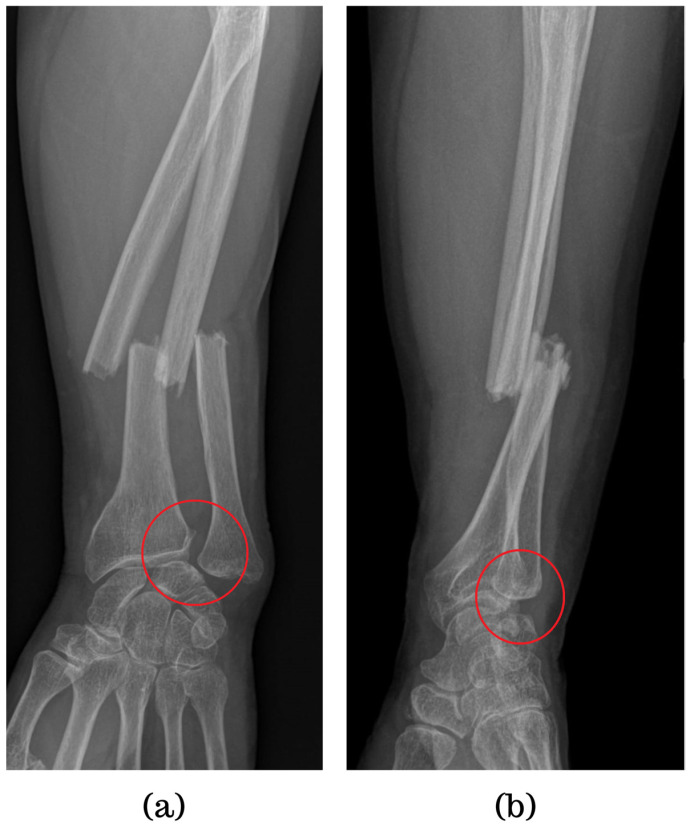
Forearm fracture with radiographic evidence indicating distal radioulnar joint (DRUJ) injury: (**a**) antero-posterior view (increased radioulnar space); (**b**) lateral view (dorsal dislocation of the ulnar head).

Conventional radiography is valuable for evaluating joint instability and excluding other potential causes of wrist pain, such as fractures or arthritis [12]. However, it primarily focuses on bone imaging and may not fully assess soft tissue structures, although sometimes, DRUJ injuries can be seen via simple X-ray, as illustrated in Figure 1, which shows a forearm fracture with an associated DRUJ injury (there is an increased radioulnar distance, as well as a dorsal dislocation of the ulnar head) (Figure 2). Dynamic joint motion cannot be evaluated with static radiographic images. In cases of suspected ligamentous injuries or more complex DRUJ pathology, additional imaging modalities like MRI or CT may be necessary for a comprehensive assessment [25,26].

### 5.2. Magnetic Resonance Imaging (MRI)

**Figure 3:**
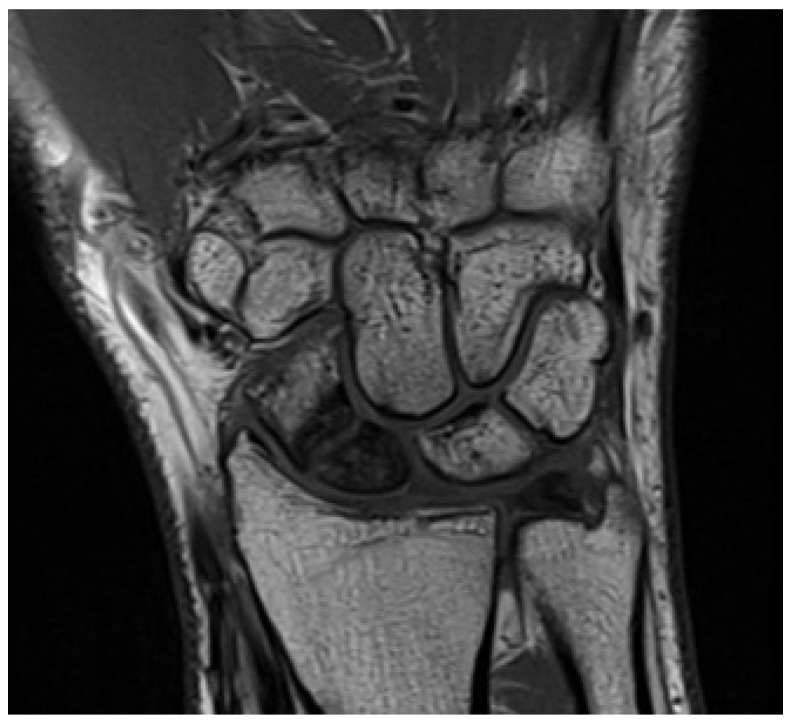
Wrist MRI evaluation of the soft tissues (intact TFCC) following a scaphoid nonunion fracture.

**Figure 4:**
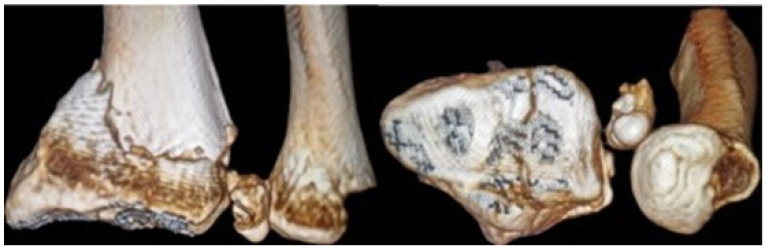
3D reconstruction of an articular distal radius fracture with associated ulnar styloid fracture displaced inside the DRUJ.

Magnetic Resonance Imaging (MRI) is the most sensitive imaging modality for diagnosing TFCC tears and associated abnormalities. Its excellent soft tissue resolution allows for accurate visualization and characterization of TFCC tears, and it is invaluable for evaluating structures involved in DRUJ stability, such as the dorsal and volar radioulnar ligaments, especially after fractures (Figure 3). MRI also helps identify bone contusions and soft tissue injuries around the joint. However, MRI has limitations for patients with contraindications like metal implants or claustrophobia and may not be as effective as radiography or CT in assessing bone-related pathologies (Figure 4). Expertise in musculoskeletal imaging is crucial for proper interpretation of MRI findings [12,25,26].

### 5.3. Ultrasound Imaging (USI)

Ultrasound Imaging (USI) is a promising, noninvasive modality for detecting translational movement in the DRUJ among healthy individuals, diagnosing interosseous membrane (IOM) tears [10], and assessing tendon disorders like tenosynovitis. USI offers real-time imaging during active range-of-motion exercises, enabling earlier detection of DRUJ instability and facilitating more effective treatment options [12,28]. USI also demonstrates high sensitivity in detecting screw protrusion following volar plating of the distal radius and aids in diagnosing associated tendon disorders [29,30].

### 5.4. Dynamic Imaging Techniques (Stress Radiography and 4D-CT)

Dynamic imaging techniques (stress radiography and 4D-CT) offer valuable insights for the evaluation of DRUJ instability during active range-of-motion exercises. Dynamic CT (4D-CT) captures multiple images of the joint during motion, generating a dynamic 3D representation that provides detailed imaging of the carpal bones and ligaments, identifying subtle abnormalities not visible on static images and playing a crucial role in surgical planning and treatment decisions. However, dynamic CT exposes patients to higher radiation levels than other imaging modalities and should be used with appropriate precautions [25,26,31].

### 5.5. Dual Fluoroscopic and Computed Tomography (DFIS-CT)

Dual Fluoroscopic and Computed Tomography (DFIS-CT) involves capturing dynamic fluoroscopic images and simultaneously acquiring high-resolution computed tomography scans during active wrist motion. This approach allows for real-time evaluation of carpal kinematics, providing detailed 3D imaging of the carpal bones and ligaments. Future studies with larger sample sizes and comparisons with established gold-standard imaging techniques are needed to establish the accuracy and reliability of DFIS-CT [26,32].

A summary of different imaging techniques and their applications is provided in Table 1.

While MRI remains the most useful diagnostic tool in diagnosing DRUJ instability and TFCC injuries [33,34], wrist arthroscopy stands out as the “gold standard” for identifying injuries to the TFCC (which is the main stabilizer of the DRUJ). It is the sole diagnostic tool capable of dynamically assessing the degree of instability and the healing potential of the injury, serving dual roles in both diagnosis and repair of the affected TFCC [35,36]. Wrist arthroscopy consistently demonstrates higher diagnostic accuracy than MRI [31].

## 6. Diverse Injuries Associated with the Distal Radioulnar Joint (DRUJ)

### 6.1. Isolated DRUJ Dislocation

Isolated acute dislocation of the distal radioulnar joint (DRUJ) is rare but noteworthy, with cases reported in the medical literature [19]. Unlike dislocations with fractures of the radius or distal ulna, isolated DRUJ dislocations are less common [37]. Dorsal dislocations present with a dorsal ulnar prominence and restrict supination, while volar dislocations manifest with a volar ulnar prominence and hinder pronation [19]. The DRUJ’s stability is maintained by various ligamentous structures, including the distal oblique bundle (DOB) [5]. These injuries can result from a fall on an outstretched hand or a direct blow to the wrist [7]. Athletes in sports requiring repetitive forearm movements, like tennis and golf, are at increased risk [38]. Treatment may involve closed reduction techniques under anesthesia, followed by immobilization with a splint or cast, and physical therapy [19]. In cases of instability, Kirschner wires may be inserted, or arthroscopic repair of the TFCC can be performed [39]. Due to its rarity, further research is needed for better understanding and management.

### 6.2. TFCC Injuries

The TFCC is essential for wrist stability and movement, consisting of the dorsal radioulnar ligament (DRIL), palmar radioulnar ligament (PRIL), and meniscus homologue (MH). It stabilizes the DRUJ and acts as a shock absorber [17,40].

TFCC tears can occur due to traumatic events (e.g., twisting or falls), degenerative changes, avulsion (forceful pulls), or ischemic conditions (reduced blood flow) [40]. These tears are relatively common, affecting 1–2 people per 100,000 annually, and may require surgical treatment if not healed spontaneously [19].

Foveal TFCC tears resulting from recurrent stress can lead to perforation and erosion of the ulnar carpus [41,42]. Schmitt et al. introduced the “CUP” classification for TFCC lesions in 2022, although it does not consider adjacent structures like the DRUJ and ulnar carpus [43]. The “iceberg concept” proposed by Atzei and Luchetti describes the TFCC’s anatomy, highlighting its distal and proximal components, and notes other structures contributing to DRUJ stability, including the ulnar collateral ligaments, extensor carpi ulnaris tendon, pronator quadratus muscle, and IOM [2,44].

Diagnostic maneuvers for foveal TFCC tears include the ulnocarpal stress test, fovea sign, DRUJ instability test, and piano-key sign [7]. Early diagnosis and treatment of TFCC injuries are crucial to prevent chronic pain, instability, and osteoarthritis [22]. Non-surgical treatment, which is often effective for patients without DRUJ instability, includes rest, immobilization, physical therapy, and NSAIDs, with a 6-month conservative period before considering surgery [22,45].

Timely management of TFCC injuries is vital for alleviating pain, restoring stability, and improving wrist function, enhancing patients’ quality of life. Healthcare professionals should prioritize early recognition and treatment, along with patient education on prevention and early reporting of wrist issues [22].

### 6.3. Ulnar Styloid Fractures

**Figure 5:**
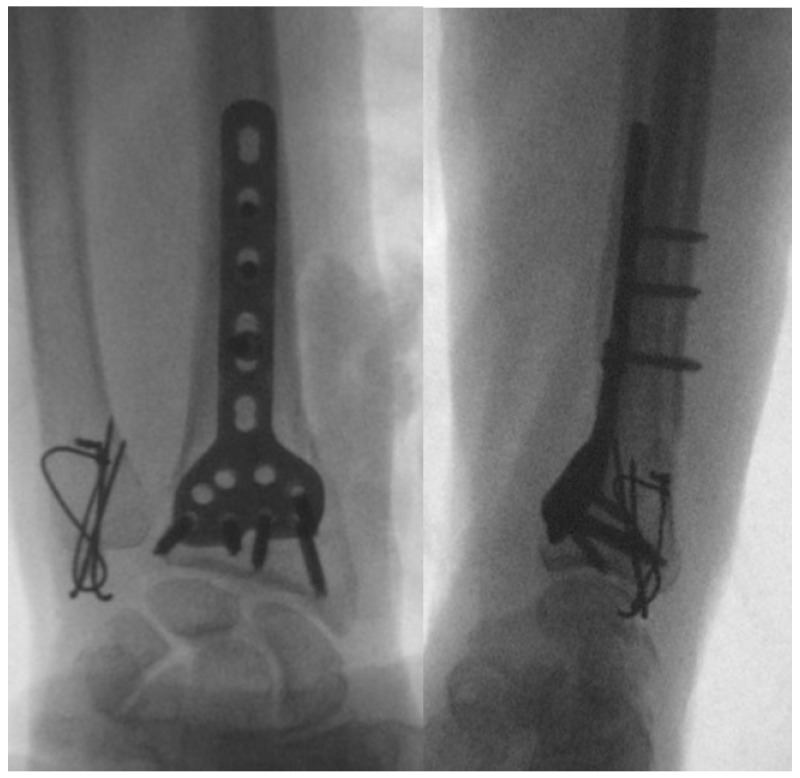
ORIF of a distal radius fracture associated with an ulnar styloid fracture.

Ulnar styloid fractures, often associated with distal radius fractures (DRFs), impact forearm rotation and stability [46]. These fractures are classified as tip and base fractures, aiding in their management [47,48]. The necessity of surgical fixation remains debated, with studies showing mixed results in terms of outcomes (Figure 5) [43,49,50]. Early detection and treatment are crucial, with nonunion repair or fragment excision sometimes being necessary [51,52].

While it was previously believed that ulnar styloid fractures might contribute to DRUJ instability, recent research suggests otherwise. The union status of the ulnar styloid, whether at the tip or base, does not affect the wrist’s range of motion or functional status. However, temporary immobilization of the DRUJ may facilitate the recovery of the TFCC [53,54].

When considering treatment options, improvements in function and symptoms can be achieved through nonunion repair or fragment excision, possibly combined with TFCC repair, tailored to the size and location of the ulnar styloid fracture [53,55]. Delayed treatment can lead to complications, such as resorption of the ulnar styloid [51]. Further research is needed to optimize management strategies [43,46,49,52].

### 6.4. Ulnar Impaction Syndrome

Ulnar impaction syndrome results from repetitive wrist loading, causing excessive pressure on the ulnar side and leading to changes primarily affecting the ulnar head and the lunate’s proximal ulnar corner [54]. This increased load can cause symptoms like ulnar abutment syndrome, proximal migration of the radius, and longitudinal instability, as often seen in Essex–Lopresti fractures [38].

Diagnosis is crucial for management and can be made using imaging techniques like X-rays or MRI scans, which identify structural abnormalities and assess severity [7]. Bone scans are also valuable when combined with radiographic assessments [56].

Treatment depends on the condition’s extent and impact on wrist function. Conservative management may involve rest, immobilization, and anti-inflammatory medication [7]. Severe cases with persistent symptoms may require surgical intervention. The main surgical options are ulnar-shortening osteotomy and the wafer procedure, performed either openly or arthroscopically [56]. These procedures reduce ulnar side load and restore wrist stability [7]. Early and accurate imaging is essential in planning treatment and preventing long-term joint complications.

### 6.5. Distal Radius Fractures

Distal radius fractures (DRFs) are common, with a significant percentage leading to DRUJ instability and symptomatic wrist pain [41]. Proper reduction and positioning of hardware during initial treatment are crucial, with immobilization or surgical intervention sometimes being necessary [8]. Intra-articular DRFs are often associated with ligamentous injuries, contributing to DRUJ instability [57]. Comprehensive evaluation and management of these fractures are essential to prevent complications and ensure optimal recovery.

### 6.6. Essex–Lopresti Injuries

Essex–Lopresti injuries involve a fracture of the radial head, dislocation of DRUJ, and rupture of IOM, leading to forearm instability [7,38]. These injuries often result from high-energy impacts, such as falls on an outstretched hand, and affect the proximal, middle, and distal radioulnar joints [38,58].

Estimates suggest the Essex–Lopresti Lesions (ELLs) occur in about 1% of radial head fractures, although recent evidence indicates that the prevalence might be closer to 5% [59]. Diagnosis is crucial for prompt management and can be made using X-rays or MRI scans, which reveal fractures, dislocations, and ligament ruptures [7,58]. Clinical signs, such as DRUJ widening or radial head migration, also aid in diagnosis [58].

Treatment typically involves surgery to restore stability and function. Procedures may include radial head replacement, DRUJ stabilization, and IOM repair or reconstruction [7,59]. In chronic cases of longitudinal radioulnar dissociation (LRUD), central band reconstruction of the IOM may be necessary [38].

Early and accurate diagnosis of Essex–Lopresti injuries is essential due to their complexity and potential long-term consequences. Prompt management improves outcomes and prevents disability. Healthcare professionals should maintain high suspicion in radial head fracture cases and use a multidisciplinary approach for optimal care [38,58,60].

### 6.7. Galeazzi Fractures

Galeazzi fractures involve a fracture of the radius and dislocation of DRUJ [7,61], comprising about 7% of forearm fractures and often resulting from high-energy trauma [11]. Symptoms include forearm swelling and deformity [62]. Various classification systems categorize these fractures based on their location relative to the DRUJ and radial styloid involvement [63,64,65]. In adults, radial shaft fractures with distal ulna fractures are termed Galeazzi equivalents [62].

**Figure 6:**
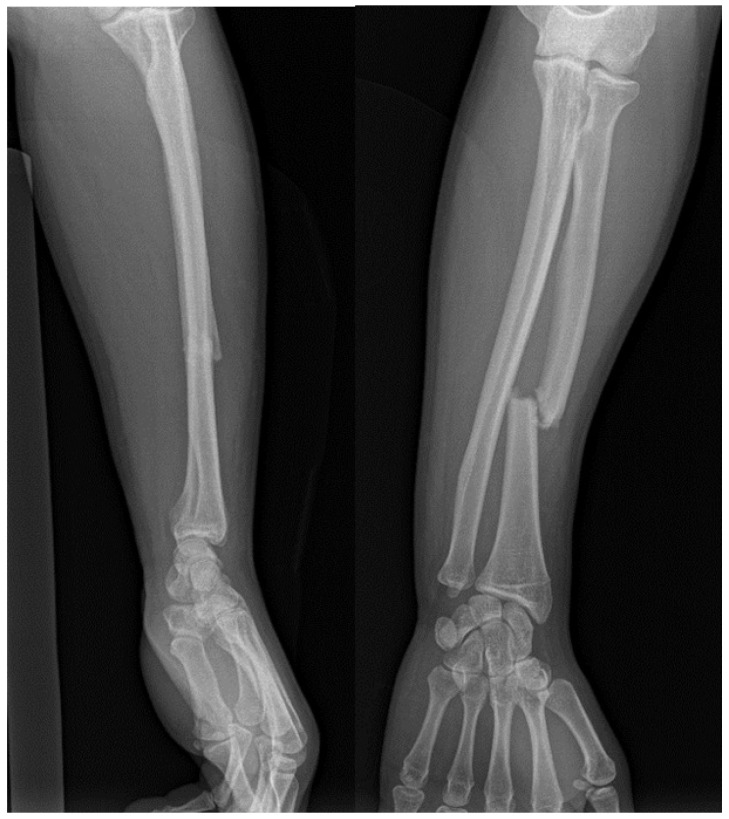
AP and lateral views of a Galeazzi fracture.

Accurate diagnosis relies on X-rays or CT scans to evaluate the DRUJ and detect neurovascular damage, although radiographic predictors of instability may not always be reliable (Figure 6) [7,62].

**Figure 7:**
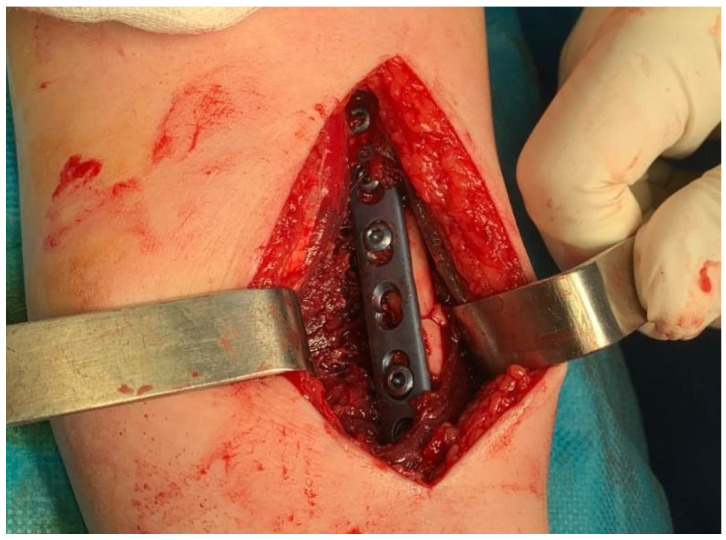
ORIF of a Galeazzi fracture via the Henry approach using a titanium plate.

Treatment typically involves surgical intervention, such as open reduction and internal fixation (ORIF) and DRUJ stabilization (Figure 7) [7,65,66]. Postoperative care includes immobilization and rehabilitation, depending on DRUJ stability [62,65,67]. Complications can include pain, stiffness, instability, malunion, nonunion, and rare neurovascular damage [58,61,62].

Prompt and appropriate treatment, often involving surgery, is crucial for optimal outcomes and preventing long-term complications. A multidisciplinary approach involving orthopedic specialists and radiologists is essential for comprehensive care and recovery [7,61,62,68].

### 6.8. Criss-Cross Injury

Classically described as simultaneous dislocation of the radial head and DRUJ without fracture [69], criss-cross injury may also involve various fractures and/or convergent elbow dislocation [70,71]. Named for the distinctive criss-cross appearance on lateral radiographs due to ulna and radius displacement, it was first described by Leung et al. in 2002 [63]. This rare injury [69] is classified by Leung et al. into the following two types: type I (anterior displacement of radial and ulnar heads at the PRUJ and DRUJ) and type II (posterior displacement of radial and ulnar heads at the PRUJ and DRUJ) [68].

Treatment involves reducing both the PRUJ and DRUJ by closed reduction or surgery, followed by early rehabilitation. Most patients regain good forearm function with either conservative or surgical treatment [70].

### 6.9. Chronic Instabilities and DRUJ Arthritis

Chronic instability of the DRUJ can lead to joint degeneration and arthritis, often triggered by trauma, rheumatoid arthritis, or congenital abnormalities [17,72]. DRUJ stability is crucial for grip strength and forearm movement and function, relying on joint surface alignment and soft tissue integrity within the TFCC, DRUJ capsule, and IOM [12].

Instability can cause ulnar impaction syndrome due to repetitive wrist loading, leading to ulnar lengthening and joint wear [7,43]. Improperly managed Galeazzi fractures and Essex–Lopresti injuries can also result in chronic DRUJ instability, potentially leading to arthritis [61,62,66].

**Figure 8:**
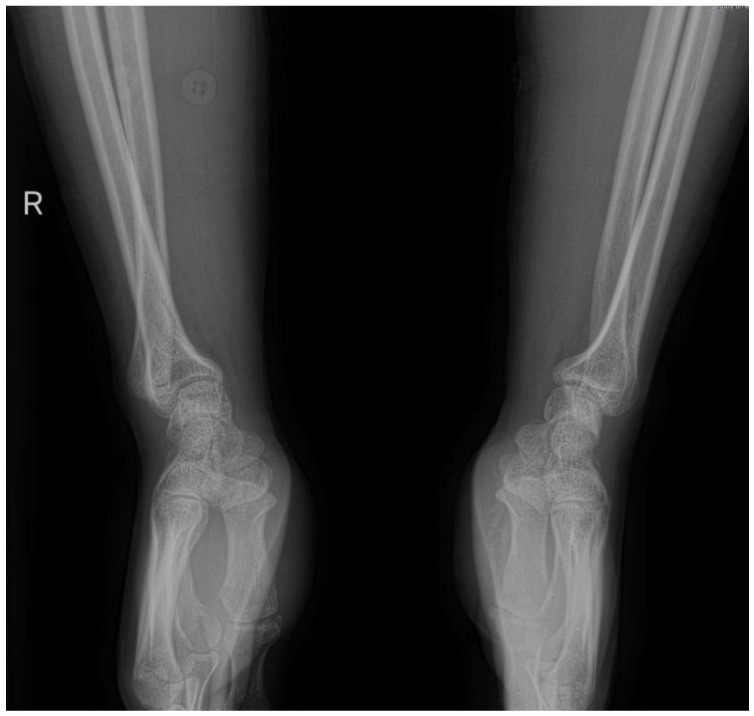
Dorsal dislocation of the right ulnar head following a distal radius fracture managed with closed reduction and immobilization.

Diagnosis involves imaging techniques like MRI or arthroscopy (Figure 8) [7,66]. Treatment options include conservative management or surgical intervention, such as DRUJ arthroplasty or arthroscopy. There is no single universally accepted treatment algorithm, so individualized plans are necessary [64,65,72].

In conclusion, chronic DRUJ instability can lead to joint degeneration and arthritis, highlighting the importance of early and proper management. A comprehensive, multidisciplinary approach is essential for effective treatment and joint preservation [61,62,72].

An overview of these injuries, including their mechanisms, diagnostic approaches, treatment options, and prevalence/incidence rates is shown in Table 2.

## 7. Treatment

In addressing DRUJ instability, it is crucial to understand the anatomical foundations of DRUJ stability and utilize clinical and paraclinical methods for evaluation [5].

The severity of the injury guides treatment. For mild to moderate DRUJ injuries, non-surgical approaches are common. These include activity modification, immobilization in a cast or brace, and physical therapy to reduce symptoms and improve joint stability [5,17,74]. External wristband braces are effective in stabilizing the DRUJ during various forearm rotations, reducing volar and dorsal translations [74]. Non-surgical methods are also the primary treatment for all types of TFCC injuries [75,76].

Severe cases may require surgical interventions to restore stability and function. Surgical options include repair (open or arthroscopic) or reconstruction of the TFCC or DOB, ulnar shortening osteotomy, and arthrodesis [5,22,75,76]. The choice of surgical treatment depends on the injury’s specifics and the patient’s overall health.

**Figure 9:**
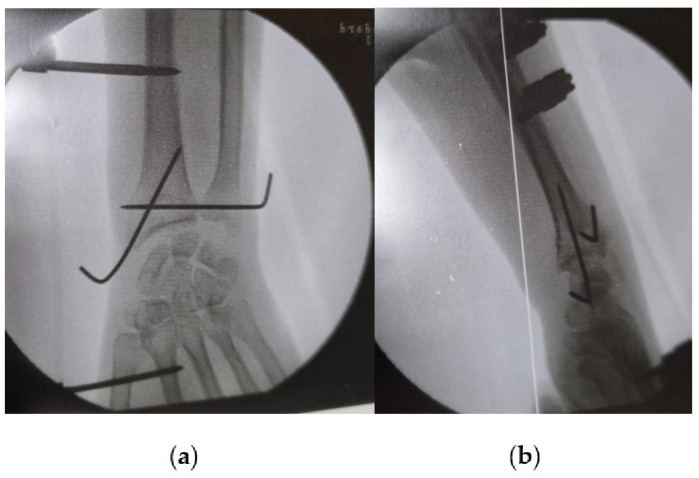
Intraoperative images of DRUJ pinning after distal radius fracture fixation with K wires and an external fixator: (**a**) antero-posterior view; (**b**) lateral view.

When it comes to DRUJ instability associated with fractures, after the surgical fixation of the main fracture, DRUJ pinning is an option for severe or chronic injuries, although it carries a higher risk of complications (Figure 9) [60]. Immobilization is suitable for milder injuries or patients not fit for surgery. The choice depends on factors like injury severity, chronicity, patient health, surgeon experience, and patient preferences. An experienced orthopedic surgeon should evaluate to determine the most appropriate approach for Essex–Lopresti injuries [59,60].

According to recent research, trans fixation of the DRUJ should be conducted in a position where optimal reduction is attainable. Based on the findings of a recent study, this position typically corresponds to supination [67].

Gaspar et al. proposed a treatment involving the reconstruction of the IOM with the Mini TightRope Device^®^ (Arthrex, Inc., Naples, FL, USA), ulnar shortening osteotomy, and simultaneous arthroscopic TFCC repair, reporting improvements in motion, grip strength, and QuickDASH scores [77]. Reconstructing the central band (CB) in Essex–Lopresti injuries restores stability, reduces distal ulnar impaction forces, and alleviates stress on the radio-capitellar joint, although complications can occur [38,58].

Both open and arthroscopic TFCC repair is used with success, achieving similar outcomes in term of DRUJ stability [78]. Most techniques utilize micro-sutures anchors or trans-osseous sutures, as well as rein-type capsular sutures, with similar functional results achieved across all techniques [28,76,78,79].

Arthroscopy is a minimally invasive surgical procedure that can be used to directly visualize the TFCC [40,78,80]. Recent anatomical studies have highlighted the proximity of the transverse branch of the dorsal branch of the ulnar nerve to the portals used in DRUJ arthroscopy, raising concerns about safety [81,82]. However, the advantage of DRUJ arthroscopy is that it allows for the visualization of the deep parts of the TFCC and its insertion area in the fovea, which cannot be adequately visualized with other methods [76,82]. This expanded visualization capability has increased the potential applicability of DRUJ arthroscopy.

Arthroscopically assisted reduction of dislocated or subluxated DRUJ has shown promising results, with minimal complications compared to traditional open reduction techniques [17]. Arthroscopy also affords the opportunity to identify and subsequently address associated soft tissue injuries, particularly to the scapholunate interosseous ligament (SLIL), lunotriquetral interosseous ligament (LTIL), and TFCC, which are common in intra-articular distal radius fractures [57].

Despite initial concerns regarding the proximity of the ulnar nerve during DRUJ arthroscopy, recent anatomical studies underscore its advantages in visualizing the TFCC and its foveal insertion. However, outcomes of arthroscopic debridement for TFCC tears may vary based on the tear type and ulnar-plus variance, potentially affecting postoperative satisfaction [25]. A recent study comparing the outcomes of suture anchor and rein-type capsular suture TFCC repair showed that the two techniques achieve similar results with satisfactory outcomes [28].

DRUJ arthroplasty involves replacing the joint with a prosthesis and is indicated for patients with severe DRUJ instability who have not responded to other treatments [41,83]. There are two main types of DRUJ prosthesis, namely total DRUJ replacement and hemiarthroplasty. The choice of prosthesis depends on the patient’s individual anatomy and needs. Total DRUJ arthroplasty distinguishes itself from other resection arthroplasties and ulnar head replacements by replacing all elements of the DRUJ, including the ulnar head, TFCC, and sigmoid notch [83]. While DRUJ arthroplasty can provide relief from pain and improve the range of motion, patients should be aware of potential complications, such as infection, nerve damage, and prosthesis loosening [41]. The use of a DRUJ arthroplasty prosthesis is a viable alternative to other DRUJ salvage procedures [83].

Several “palliative” surgical procedures are available to address ulnar head pathology, including the Darrach procedure, hemiresection procedures, the Sauvé–Kapandji procedure, and total ulnar head arthroplasty. However, these procedures may have drawbacks, such as altering the relationship with the DRUJ or leading to an unstable ulnar stump despite soft tissue stabilization procedures [83,84] (Table 3).

Rehabilitation after surgical treatment of DRUJ injuries typically involves a period of immobilization followed by gradual range-of-motion exercises and strengthening exercises to restore function and prevent recurrence [17,22,45,84].

Treating DRUJ instability requires a comprehensive approach considering injury severity, patient factors, and available non-surgical and surgical options. Conservative treatments are effective for mild to moderate cases, while severe cases may necessitate surgical interventions. Recent advances in arthroscopic techniques offer promising outcomes with less postoperative pain and faster recovery. IOM and CB reconstructions in Essex–Lopresti injuries provide significant benefits, but potential complications must be considered. Ultimately, the combination of conservative measures, surgical interventions, and rehabilitation is vital for the restoration of joint stability and function.

## 8. Suggested New Classification of DRUJ Instability by the Authors

The management of distal radioulnar joint (DRUJ) injuries is complicated by the joint’s complex anatomy and the involvement of multiple stabilizing structures. Existing treatment algorithms, like the “Four-Leaf Clover” approach [85], focus on addressing all relevant components but do not provide a straightforward classification system for clinical use. Current classifications, such as those proposed by Palmer and Atzei, primarily address specific injuries, like TFCC lesions, and lack comprehensive coverage of the full spectrum of DRUJ pathologies, particularly those involving extrinsic stabilizers like the interosseous membrane and pronator quadratus [44,86]. This limitation can lead to inconsistencies in diagnosis and treatment, especially in complex cases. Moreover, the variability in osseous anatomy and the intricate nature of soft-tissue stabilizers pose additional challenges in applying these classifications uniformly across patient populations [87]. Therefore, there is a clear need for a more practical classification system that integrates the latest understanding of DRUJ biomechanics and pathology, facilitating better diagnostic and treatment outcomes [86,87].

**Figure 10:**
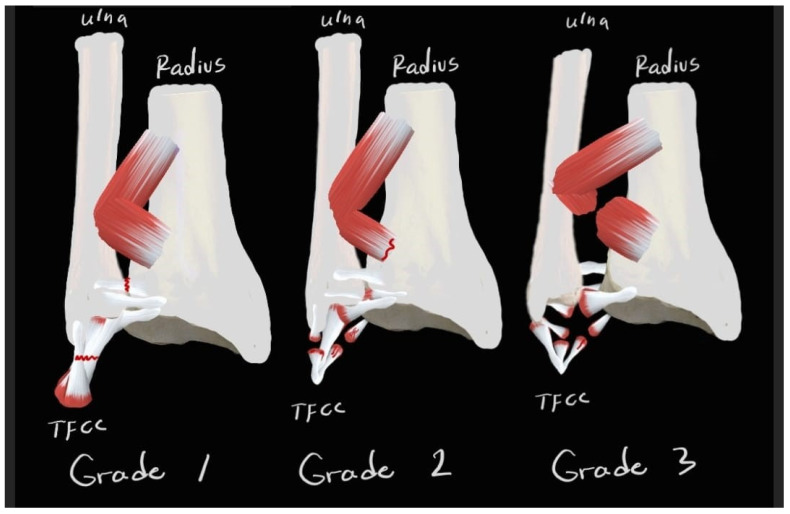
Illustration of DRUJ injury classification. Grade 1 shows normal joint alignment, with intact ligaments. Grade 2 displays subluxation and partial ligament tears. Grade 3 depicts severe dislocation with complete tears of the TFCC, as well as the DOB.

Based on our review and existing literature, we devised this classification system to improve the diagnostic approach for DRUJ injuries, integrating clinical evaluations and imaging methods. This system aims to streamline treatment decisions, as detailed in Table 4 and demonstrated in Figure 10. This classification can be applied using basic clinical and X-ray images, along with advanced imaging techniques, to accurately determine the severity of the injury.

## 9. Discussion and Future Stances

DRUJ instability and TFCC pathologies pose significant challenges in the field of orthopedic medicine. Over the years, efforts have been made to better understand the anatomical foundations of DRUJ stability and develop effective treatment approaches. In this academic discussion, we delve into the latest developments in the management of DRUJ instability, with a particular focus on emerging technologies and innovative approaches that promise to revolutionize the diagnosis, treatment, and overall outcomes of patients with DRUJ instability and TFCC injuries.

One notable advancement in the management of DRUJ instability is alternative classification systems for forearm fracture–dislocations, such as the three-locker system classification, which aims to differentiate between uncomplicated dislocations and severe dislocations that involve the forearm joint [11,61,85]. By focusing on dislocations or fracture–dislocations affecting two or more lockers of the forearm joint, this classification system provides a more intricate categorization of forearm fracture–dislocations, enhancing the understanding of various injury patterns and enabling tailored treatment approaches. This advancement is crucial in guiding clinicians to adopt a more nuanced and personalized approach when managing DRUJ instability cases [11].

In the context of evaluating the TFCC using imaging techniques, ultrasonography (US) has emerged as a promising modality [12]. US complements other imaging modalities such as radiography and MRI, offering high inter-observer reliability in detecting translational movement of the DRUJ in healthy individuals. This suggests the potential application of US in future musculoskeletal assessments and highlights its utility in accurately diagnosing and assessing DRUJ instability.

Moreover, the integration of automated 3D methods for quantifying DRUJ morphology has shown promise in enhancing surgical planning and treatment outcomes for various joints in the body [27]. By leveraging these advanced imaging technologies, clinicians can gain a deeper understanding of the complex anatomical structures involved in DRUJ instability and TFCC injuries, leading to more precise and effective treatment decisions.

Innovative imaging techniques have also addressed the challenges encountered when evaluating the TFCC using grayscale ultrasound. Power Doppler, a specialized ultrasound technique that detects blood flow, has proven valuable in improving the evaluation and diagnosis of TFCC injuries [88]. By assessing the presence of increased vascularity at the deep border of the ulnocarpal ligament and the outer margin of the TFCC, along with other indications, such as joint widening and visible gaps, clinicians can enhance diagnostic accuracy. Additionally, magnetic resonance arthrography (MRA) has emerged as another valuable imaging tool in assessing TFCC injuries [89,90]. MRA provides detailed images of the TFCC’s structure and its relationship with surrounding tissues, aiding in identifying subtle tears and occult injuries that may not be apparent in conventional MRI.

The incorporation of these innovative imaging techniques and technologies in the assessment of DRUJ and TFCC conditions holds great promise for advancing the field of musculoskeletal diagnostics and treatment planning. The ability to accurately visualize and assess these complex structures will undoubtedly lead to better-informed treatment decisions and improved patient outcomes.

In addition to advancements in imaging, research into minimally invasive techniques for DRUJ conditions is gaining momentum. Emerging methods such as arthroscopy-assisted procedures and percutaneous fixation techniques are being explored to offer less invasive and efficient treatment options. Arthroscopic surgery continues to gain popularity as an effective treatment option for DRUJ instability, offering advantages like minimal invasiveness, reduced soft tissue damage, and quicker recovery. Anatomical reconstruction of the distal radioulnar ligaments (DRULs) through a tunnel created between the ulnar head and the sigmoid notch of the radius, along with arthroscopic repair of the TFCC, has shown promising outcomes, with the potential to enhance patient recovery and overall treatment success [91,92]. These advancements pave the way for better management of DRUJ conditions and improved patient outcomes.

Furthermore, wrist arthroscopy performed under local anesthesia without the need for a tourniquet and sedation presents a promising strategy for addressing DRUJ issues [89]. This approach offers several advantages, including reduced risks and costs associated with the procedure. Patients can remain conscious throughout the surgery, avoiding potential complications related to general anesthesia or regional blocks. The absence of a tourniquet enhances safety by minimizing the risk of nerve injury, skin necrosis, and postoperative discomfort. Moreover, wrist arthroscopy performed under local anesthesia allows patients to provide real-time feedback to the surgeon, aiding in accurate diagnosis and treatment of DRUJ problems. With positive outcomes and high patient satisfaction, this technique shows potential as an effective and cost-efficient option for managing DRUJ conditions, leading to its potentially wider adoption in clinical settings.

In conclusion, the field of orthopedics has witnessed remarkable advancements in the management of DRUJ instability and TFCC pathologies. These developments, ranging from novel classification systems to innovative imaging techniques and minimally invasive approaches, are transforming the approach to diagnosis and treatment. As emerging technologies continue to shape the field, the future of managing these challenging wrist injuries looks brighter than ever before.

It is essential to acknowledge that while these advancements hold immense potential, further research and validation are necessary to establish their widespread clinical applicability. Collaborative efforts among researchers, clinicians, and industry partners are critical to continue pushing the boundaries of orthopedic care and bringing these innovations to patients globally. Additionally, the cost-effectiveness and accessibility of these advanced technologies must be carefully considered to ensure equitable distribution and utilization in healthcare settings worldwide.

As the field of orthopedics continues to evolve, the combined efforts of clinicians, researchers, and technology developers will drive forward the frontiers of orthopedic care, ultimately benefiting patients and transforming the landscape of wrist injury management. With a comprehensive understanding of the anatomical foundations, combined with cutting-edge technologies and less invasive approaches, the future holds great promise for improved outcomes and enhanced quality of life for patients facing these complex wrist injuries. The evolving landscape of orthopedic medicine presents an exciting era of innovation and progress in the management of DRUJ instability and TFCC pathologies. As ongoing research and technological advancements continue to shape the field, the future of managing these challenging wrist injuries looks brighter than ever before.

## 10. Conclusions

The management of DRUJ instability remains complex, involving both conservative and surgical treatments. This review provides a thorough overview of DRUJ instability, examining various treatment approaches and future directions in the field.

Accurate evaluation of DRUJ instability requires understanding of its anatomical basis and the employment of precise diagnostic methods. Advanced imaging techniques such as high-resolution MRI and 3D-CT scanning have enhanced diagnostic accuracy and surgical planning [12,17,27]. The revised classification system offers a more comprehensive approach to complex forearm fracture–dislocations, moving beyond traditional eponyms [17,61].

Conservative treatments, including activity modification, immobilization, and physical therapy, are effective for mild to moderate cases [5,17]. Severe cases may necessitate surgical interventions, such as TFCC repair, ulnar shortening osteotomy, or arthrodesis, tailored to the individual patient’s needs [5,23]. Recent advancements in arthroscopy provide benefits like reduced invasiveness and faster recovery [79,89]. Additionally, wrist arthroscopy under local anesthesia offers a promising approach, improving patient safety and allowing for real-time feedback [91].

Procedures like IOM and CB reconstruction for Essex–Lopresti injuries aim to restore stability but carry risks such as iatrogenic fractures and nerve injury. Future research should focus on optimizing these techniques and exploring new strategies [38].

Overall, integrating advanced imaging and arthroscopic techniques, along with ongoing technological innovations, holds promise for better management of DRUJ instability. Continued advancements are expected to improve patient outcomes and care quality.

## Figures and Tables

**Table 1:** Comparison of imaging techniques for DRUJ instability evaluation.

Imaging Technique	Key Advantages	Applications
Conventional radiography	High-quality bone imaging; Cost-effective and widely available; Quick acquisition for emergency situations [12].	Evaluation of fractures, dislocations, and degenerative changes in the DRUJ [12,25].
Magnetic resonance imaging (MRI)	Excellent soft tissue resolution; Differentiation of acute from chronic injuries; Non-invasive and preferred for soft tissue assessment [24].	Diagnosis of TFCC tears and associated abnormalities; Assessment of structures involved in DRUJ stability; Identification of bone contusions and soft tissue injuries [12,24].
Ultrasound imaging (USI)	Real-time imaging during active movements [12]; Detection of TFCC tears with high sensitivity [27]; Ability to detect screw protrusion post volar plating [30].	Detection of translational movement in the DRUJ among healthy individuals [27]; Diagnosis of interosseous membrane (IOM) tears [10]; Assessment of tendon disorders, such as tenosynovitis [29].
Dynamic imaging techniques (stress radiography and 4D-CT)	Provides insights during active range-of-motion exercises [24]; Offers real-time assessment of carpal kinematics; Enables comprehensive evaluation of carpal bones and ligaments [25].	Evaluation of DRUJ instability during active range-of-motion exercises; Identification of subtle abnormalities not visible in static images; Surgical planning and treatment decisions [24,25].
DFIS-CT (dual fluoroscopic and computed tomography)	Captures dynamic fluoroscopic and CT images; Real-time evaluation of carpal kinematics [25,31].	In vivo assessment of 3D carpal kinematics; Evaluation of SLIL repair failure and other carpal abnormalities; Potential low-dose alternative for wrist kinematics assessment [25,31].

**Table 2:** Main injuries associated with DRUJ Instability.

Type of Injury	Mechanism	Diagnosis and Imaging	Treatment	Prevalence/Incidence
Isolated DRUJ dislocation	Fall on outstretched hand or direct blow to the wrist	Clinical presentation: dorso-, or volar–ulnar prominence, restricted supination or pronation. Imaging: X-rays, MRI [18,36].	Closed reduction techniques, immobilization with splint or cast, physical therapy. Surgical options: Kirschner wire insertion, arthroscopic repair of TFCC [18,38].	Rare; less common than dislocations with fractures [36].
TFCC injuries	Traumatic, degenerative avulsion, ischemic tears	Clinical presentation: wrist pain and dysfunction. Imaging: X-rays, MRI, bone scans. Physical testing maneuvers: ulnocarpal stress test, fovea sign, etc. [7,39].	Conservative management: rest, immobilization, NSAIDs, physical therapy [21]. Surgical options: arthroscopic or open repair of TFCC [44].	Incidence: 1–2 per 100,000 people per year [18].
Ulnar styloid fractures	High-energy trauma, falls on outstretched hand	Clinical presentation: forearm rotation and stability affected. Imaging: X-rays, CT scans [45].	Classification and diagnosis based on location and pattern type [47]. Treatment options: conservative management, surgical fixation, ulnar shortening osteotomy [45,51].	21–61% of DRFs have ulnar styloid fractures [46].
Ulnar impaction syndrome	Repetitive wrist joint loading	Imaging: X-rays, MRI. Bone scans may also be used [7,54].	Conservative management: rest, immobilization, anti-inflammatory medication [7]. Surgical intervention: ulnar shortening osteotomy, wafer procedure [73].	Common cause of ulnar sided wrist pain [73].
Distal radius fractures	Falls, high-energy trauma	Clinical presentation: wrist pain, reduced range of motion. Imaging: X-rays, CT scans [8].	Management: proper reduction, immobilization, surgical intervention when necessary [8]. Complications: associated with DRUJ instability and ligamentous injuries [40,56].	Common injuries: DRUJ instability in 2–37% of cases [40].
Essex–Lopresti injuries	High-energy loads applied axially to the forearm	Imaging: X-rays, MRI scans. Clinical signs: DRUJ widening, radial head migration, subluxation of ulna [7].	Surgical procedures: radial head replacement, DRUJ stabilization, interosseous ligament repair or reconstruction [7,59], multidisciplinary approach for optimal care [37].	Estimated prevalence: 1% to possibly as high as 5% [58].
Galeazzi fractures	High-energy trauma	Clinical presentation: forearm swelling, deformity [60]. Imaging: X-rays, CT scans [7].	Surgical intervention: ORIF, DRUJ stabilization [7,65,66], postoperative care and rehabilitation. Complications may include persistent pain, stiffness, instability [60].	Comprises approximately 7% of all forearm fractures [61].
Criss-cross injury	Simultaneous dislocation of radial head and DRUJ	Diagnosis: imaging (X-rays, MRI) [68,70]. Classification: types I and II based on displacement direction [67].	Treatment: closed reduction or surgery, early rehabilitation. Good prognosis with either conservative or surgical treatment [69].	Extremely rare injury [68].
Chronic DRUJ instabilities	Traumatic injuries, rheumatoid arthritis	Diagnosis: imaging (MRI, arthroscopy). Treatment: conservative management or surgical intervention (arthroplasty, arthroscopy), multidisciplinary approach [17,71].	Chronic instability can lead to joint degeneration and ulnar impaction syndrome. Treatment aims to preserve joint health and function [71].	Associated with various conditions [17,71].

**Table 3:** Palliative surgical procedures for DRUJ.

Bower’s Procedure	This method includes the removal of a significant part of the ulnar head while keeping the ulnar styloid, TFCC, and ulnar cortical column intact. It ensures continuity between the ulnar column and radius, providing support from the ulnar styloid and TFCC.
Sauvé–Kapandji Procedure	This technique preserves stability on the ulnar aspect of the joint by keeping the ulnar head intact and securing it with screws to the ulnar notch of the radius. To restore forearm rotation, a segment of the ulna is removed at the ulnar neck.
Darrach’s Procedure	Darrach’s procedure involves removing the distal portion of the ulna. It was initially described by William Darrach in 1912 for the treatment of distal radius pseudarthrosis. The recommended excision site is at the upper end of the ulnar notch.

**Table 4:** DRUJ injury classification.

Grade	Imaging Findings	Clinical Presentation	Treatment
Grade 1	- X-ray/CT: normal joint congruency - US/MRI: ligamentous integrity may appear intact	- Occasional discomfort or mild limitation of wrist movement	- Conservative management: - Activity modification - Immobilization - Physical therapy
Grade 2	- X-ray/CT: joint subluxation or incongruity may be present in stress tests or 4D-CT scans - US/MRI: Partial ligamentous tears or attenuations (TFCC/DOB/volar and dorsal radioulnar ligaments)	- Persistent pain, occasional clicking or catching sensations, mild to moderate limitation of wrist movement	- Conservative management: - Immobilization - Physical therapy - Possible surgical intervention (DRUJ pinning, TFCC repair) depending on severity and chronicity of symptoms.
Grade 3	- X-ray/CT: severe joint incongruity/dislocation - US/MRI: complete ligamentous tears (complex TFCC, DOB, and pronator quadratus tears) and joint dislocation	- Persistent pain, significant functional impairment, instability during daily activities - Possible signs of nerve compression or vascular compromise.	- Surgical intervention: Repair or reconstruction of ligaments (TFCC and DOB), ulnar shortening osteotomy, arthrodesis, or DRUJ arthroplasty depending on injury and patient’s health.

## Data Availability

Not applicable.
